# Classification of Obsessive-Compulsive Disorder Using Distance Correlation on Resting-State Functional MRI Images

**DOI:** 10.3389/fninf.2021.676491

**Published:** 2021-10-20

**Authors:** Qian Luo, Weixiang Liu, Lili Jin, Chunqi Chang, Ziwen Peng

**Affiliations:** ^1^School of Biomedical Engineering, Health Science Center, Shenzhen University, Shenzhen, China; ^2^Guangdong Key Laboratory for Biomedical Measurements and Ultrasound Imaging, Shenzhen University, Shenzhen, China; ^3^National-Regional Key Technology Engineering Laboratory for Medical Ultrasound, Shenzhen University, Shenzhen, China; ^4^Guangdong Key Laboratory of Mental Health and Cognitive Science, South China Normal University, Guangzhou, China; ^5^Center for Studies of Psychological Application, School of Psychology, South China Normal University, Guangzhou, China; ^6^Peng Cheng Laboratory, Shenzhen, China; ^7^Department of Child Psychiatry, Shenzhen Kangning Hospital, Shenzhen University School of Medicine, Shenzhen, China

**Keywords:** obsessive-compulsive disorder, functional connectivity, distance correlation, classification, rs-fMRI

## Abstract

Both the Pearson correlation and partial correlation methods have been widely used in the resting-state functional MRI (rs-fMRI) studies. However, they can only measure linear relationship, although partial correlation excludes some indirect effects. Recent distance correlation can discover both the linear and non-linear dependencies. Our goal was to use the multivariate pattern analysis to compare the ability of such three correlation methods to distinguish between the patients with obsessive-compulsive disorder (OCD) and healthy control subjects (HCSs), so as to find optimal correlation method. The main process includes four steps. First, the regions of interest are defined by automated anatomical labeling (AAL). Second, functional connectivity (FC) matrices are constructed by the three correlation methods. Third, the best discriminative features are selected by support vector machine recursive feature elimination (SVM-RFE) with a stratified N-fold cross-validation strategy. Finally, these discriminative features are used to train a classifier. We had a total of 128 subjects out of which 61 subjects had OCD and 67 subjects were normal. All the three correlation methods with SVM have achieved good results, among which distance correlation is the best [accuracy = 93.01%, specificity = 89.71%, sensitivity = 95.08%, and area under the receiver-operating characteristic curve (AUC) = 0.94], followed by Pearson correlation and partial correlation is the last. The most discriminative regions of the brain for distance correlation are right dorsolateral superior frontal gyrus, orbital part of left superior frontal gyrus, orbital part of right middle frontal gyrus, right anterior cingulate and paracingulate gyri, left the supplementary motor area, and right precuneus, which are the promising biomarkers of OCD.

## Introduction

Obsessive-compulsive disorder (OCD) is a mental disorder that causes repeated and unwanted thoughts and/or obsessive feelings and compulsive actions and it can limit the ability of the patient to take part in relationships, the workplace, and in society (Piacentini et al., 2003; Abramowitz et al., 2009). Its prevalence is about 1–3% lifetime (Ruscio et al., 2010; Rapinesi et al., 2019). In clinical practice, no diagnostic biomarkers are available for OCD and its diagnosis is always based on some symptom-oriented criteria according to the International Classification of Diseases (ICD; Stein et al., 2016) and the Diagnostic and Statistical Manual of Mental Disorders (DSM; Battle, 2013). However, these criteria may have several problems over the conditions of an individual. For example, the patients with OCD often co-occur with depression and anxiety or another psychiatric comorbidity, which can contribute to misdiagnosis.

With the development of medical imaging, researchers can explore the pathogenesis of OCD. Currently, the pathogenesis of OCD has been confirmed to be caused by the cortico-striato-thalamo-cortical (CSTC) circuit dysfunction, but emerging evidence indicates that broader brain regions, such as the left supplementary motor area (SMA) and right precuneus, are involved in this disorder (Saxena et al., 1998; Rehn et al., 2018; Thorsen et al., 2018; Hazari et al., 2019). These changes in the brain are due to the diversity of tasks in the investigation of OCD. Therefore, task-based functional MRI (task-fMRI) has been studied for detecting the functional changes in the brain in patients with OCD and their relatives (Menzies et al., 2008a). However, task-fMRI studies can only focus on some specific regions of the brain and may have missed important information existing in regions of the brain not related to the task. Without specific design in task-fMRI, resting-state functional MRI (rs-fMRI) provides an effective and noninvasive approach to assess the neural activation and functional connectivity (FC) of the human brain without any hypothesis. It can also provide a reliable measure of baseline brain activity and may complement and extend findings from task-based studies (Biswal et al., 1995; Hou et al., 2014; de Vries et al., 2019; Yang et al., 2019).

Recently, the multivariate pattern analysis based on a machine learning (ML) algorithm has been introduced for neuroimaging analysis of a variety of diseases such as autism, depression, and schizophrenia (Sajda, 2006; Anderson et al., 2011; Zeng et al., 2012; Liu et al., 2014, 2015a, 2017; Mueller et al., 2015; Rathore et al., 2017; Lamothe et al., 2018; Zhou et al., 2018; Bu et al., 2019; Rapinesi et al., 2019). It has the advantage of being able to inference individual level over the univariate analysis used at the group level (Orrù et al., 2012; Goodman et al., 2014). In comparison to other traditional methods of analysis, its ability to use inter-regional correlations, such as the Pearson correlation, to detect subtle and spatially distributed effects (Menzies et al., 2008b; Bruin et al., 2020; Zhan et al., 2021). Therefore, it seems particularly well-suited for the neuroimaging analyses in OCD, as abnormalities are typically distributed across the brain (Klöppel et al., 2008; Arbabshirani et al., 2017).

**Figure 1:**
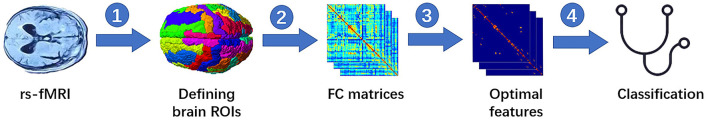
Classification of flow chart with four main steps: (1) defining the ROIs from the rs-fMRI images or by using anatomical templates that have been defined, (2) extracting rs-fMRI time series based on the ROIs and calculating the FC matrices, (3) using feature selection method to define the optimal features from the FC matrices, and (4) training a classifier. ROIs, region of interests; rs-fMRI, resting-state functional MRI; FC, functional connectivity.

In this study, we employed the multivariate pattern analysis *via* the three correlation methods to distinguish the patients with OCD from a healthy control subject (HCS). A general flowchart of rs-fMRI based on the FC matrix for diagnosis is shown in Figure 1. In this framework, there are four main steps: (1) defining the region of interests (ROIs) from the rs-fMRI images or by using the anatomically and functionally defined reference atlases of the brain, (2) extracting rs-fMRI time series based on the ROIs and calculating the FC matrices, (3) using feature selection method to get the optimal features from the FC matrices, and (4) training a classifier.

Currently, this study mainly focused on the second and third parts. In the second part, with rs-fMRI time series data, the FC matrix can be extracted for characterizing the network structure of the brain. One way is to calculate the Pearson correlation between rs-fMRI time series over the ROIs predefined as automated anatomical labeling (AAL) with 116 structural regions (Tzourio-Mazoyer et al., 2002). For example (Shenas et al., 2013; Gruner et al., 2014; Sen et al., 2016; Takagi et al., 2017), the authors use Pearson correlation as the network features. In addition, the partial correlation was also used for measuring the FC (Varoquaux et al., 2010; Smith et al., 2011; Dadi et al., 2019). However, the Pearson correlation and partial correlation only discover the linear dependency, although the partial correlation excludes the indirect influence of the correlation structure. To overcome this limitation, the distance correlation was proposed to measure both linear and non-linear associations between the two ROIs (Szekely et al., 2007; Yoo et al., 2019).

In the third part, because of high-dimensional features from the FC matrix, we need a feature selection algorithm to reduce the dimensionality. In literature, the recursive feature elimination (RFE) algorithm is a very excellent feature selection technique that has been widely used in many fields (Guyon et al., 2002; Ding et al., 2015; Liu et al., 2015b; Lin et al., 2017; Wang et al., 2019), but it needs a specific classifier. Currently, studies on the diagnosis of OCD disease are usually limited to a small data set, so the researchers tend to use traditional ML methods to complete the task. Among them, support vector machine (SVM) provides excellent performance (Shenas et al., 2013; Gruner et al., 2014; Sen et al., 2016; Takagi et al., 2017; Wang et al., 2019). Therefore, we applied the SVM-RFE algorithm to filter the features.

In the fourth part, these selected features were entered into the seven classifiers. According to the final classification performance, we can explore the optimal FC method and classifier and investigate the regions of the brain, which may be potential biomarkers. Finally, our aims were 2-fold: one is to investigate which correlation method achieves the best discrimination between OCD and HCS and the other is to explore some potential biomarkers according to the above results.

## Materials and Methods

### Participants

This study was approved by the Ethics Committee of Shenzhen Kangning Hospital and the written informed consent was obtained from each participant. A total of 128 subjects were enrolled from Shenzhen Kangning Hospital and Guangzhou Brain Hospital, including 67 HCS and 61 patients with OCD, aged from 13 to 63 years old. The demographic information and clinical characteristics information are shown in Table 1. The independent sample *t*-test was carried out on the age (*p* = 0.45). For the Yale-Brown Obsessive Compulsive Scale (Y-BOCS) total score, the Y-BOCS obsessions score, and the Y-BCOS compulsions score (*p* < 0.001), we used the independent sample Kruskal–Wallis test.

**Table 1:** Demographic and clinical characteristics of the participants.

**Variable**	**OCD**	**HCS**	***p-*value**
**Demographic measure**			
Number	61	67	-
Sex	M43, F18	M 48, F 19	-
Age average	27.7 ± 8.4	28.9 ± 8.5	*p* = 0.45
**Clinical measures**			
YBOCS total score	26.8 ± 6.1	2.3 ± 3.4	*p* < 0.001
YBOCS obsessions score	14.6 ± 3.8	1.2 ± 1.7	*p* < 0.001
YBCOS compulsions score	12.2 ± 5.0	1.2 ± 2.1	*p* < 0.001

*OCD, obsessive-compulsive disorder; HCS, healthy control subject; Y-BOCS, Yale-Brown Obsessive Compulsive Scale*.

### Imaging Data Acquisition

A 3.0-Tesla MR system (Philips Medical Systems, Best, the Netherlands) equipped with an eight-channel phased-array head coil was used for the data acquisition. Functional data were collected by using gradient echo-planar imaging (EPI) sequences [time repetition (TR) = 2,000 ms, echo time (TE) = 60 ms, flip angle = 90°, 33 slices, field of view (FOV) = 240 mm^2^ × 240 *mm*^2^, matrix = 64 × 64, slice thickness = 4 mm, and voxel size = 3.75 mm^3^ × 3.75 mm^3^ × 4 *mm*^3^]. For each participant, the fMRI scanning lasted for 480 s and 240 volumes were obtained. For spatial normalization and localization, a high-resolution T1-weighted anatomical image was also acquired by using a magnetization prepared gradient echo sequence (TR = 8 ms, TE = 3.7 ms, flip angle = 7°, FOV = 240 mm^2^ × 240 *mm*^2^, matrix = 256 × 256, slice thickness = 1 mm, and voxel size = 0.94 *mm*^3^ × 0.94 *mm*^3^ × 1 *mm*^3^). During the scanning, the participants were instructed to relax with their eyes closed and stay awake without moving.

### Data Preprocessing

The data were preprocessed by using the Statistical Parametric Mapping toolbox (SPM12, https://www.fil.ion.ucl.ac.uk/spm) and the Data Processing Assistant for Resting-State fMRI (DPARSF version 4.4, http://rfmri.org/dpabi; Shenas et al., 2013, 2014). Image preprocessing consisted of: (1) removing first the 10-time points; (2) slicing timing correction; (3) realigning the time series of the images for each subject; (4) T1-weighted individual structural images by coregistered to the mean functional image; (5) the transformed structural images by segmented into gray matter, white matter, and cerebrospinal fluid; (6) based on these segmented images, using diffeomorphic anatomical registration through exponentiated lie algebra (DARTEL) (Ashburner, 2007) tool to estimate the normalization parameters from individual native space to the Montreal Neurological Institute (MNI) space (Xue et al., 2020); (7) the functional imaging data normalized to the MNI space by using these normalization parameters and resampling at 3 *mm*^3^ × 3 *mm*^3^ × 3 *mm*^3^; (8) nuisance covariate regression (head motion parameters, white matter signal, and cerebrospinal fluid signal); (9) spatial smoothing with a 4-mm full-width half-maximum isotropic Gaussian kernel; (10) band-pass filtering (0.01–0.08 Hz); and (11) micro-head-motion correction according to framewise displacement (FD) by replacing the rs-fMRI volume with FD > 0.5 mm (nearest neighbor interpolation).

### Definition of ROIs and Calculation of the FC Matrix

In this study, we employed an AAL atlas to define the ROIs. For the calculation of the FC matrix, the Pearson correlation, partial correlation, and distance correlation methods will be used in this study. For each subject, the mean of time series over all voxels in each region was extracted. The FC matrices were calculated between these average time courses with the three correlation methods implemented with Nilearn software (http://nilearn.github.io/). Considering that the matrix was symmetric, we only needed to take the lower triangle of the matrix. Finally, we flattened the lower trig matrix to get a feature vector with a length of (116 × 116 – 116)/2 = 6,670. In our following experiment, each feature was normalized by Fisher's *z*-transformation (Fisher, 1915; Vergun et al., 2013; Kassraian-Fard et al., 2016).

### Feature Selection and Classification

In this study, to reduce the dimension of the data and find the most discriminative subset of feature, we applied SVM-RFE with a stratified N-fold cross-validation strategy for feature selection (SVM-RFE-NCV). It is a sequential backward selection algorithm based on the maximum margin principle of SVM under the N-fold cross-validation. The process contains five steps: (1) training the model with the samples, (2) sorting the scores of each feature, (3) removing the features with the minimum scores, (4) training the model again with the remaining features and repeating the process, and (5) selecting the required features (Ding et al., 2015; Wang et al., 2019). With the selected features, seven classifiers are compared: SVM with linear kernel, multilayer perceptron (MLP), extreme gradient boosting (XGBoost), gradient boosting decision tree (GBDT), graph convolution network (GCN), and sparse L1 and non-sparse L2 regularization for the logistic regression classifiers (LR-L1 and LR-L2; Friedman et al., 2001; Chen and Guestrin, 2016; Kipf, 2017). The SVM-RFE-NCV process was embedded in a classification framework with 10-fold cross-validation (10-CV).

### Performance Evaluation

The performance of the proposed classifiers is assessed by using the four performance measures: specificity, sensitivity, accuracy, and area under the receiver-operating characteristic curve (AUC). To test whether these classification scores are significant, we performed a permutation test: we first randomly reassigned the subject labels and then performed the 10-CV classification. This procedure was repeated by 1,000 times. The *p*-value was then calculated by dividing the number of times that showed a higher value than the derived from the non-permuted model by the total number of permutations (Plitt et al., 2015).

## Results

In this study, some qualitative and quantitative comparison results are provided. At first, we qualitatively compared the three methods *via* the scatter plot and correlation visualization. Then, the classification results of the OCD and HCS are evaluated according to the pipelines composed of the three correlation measures, the SVM-RFE-NCV, and the seven classifiers, to select correlation measure and classifier to obtain the best discrimination between the OCD and HCS. In addition, we will use the SVM-RFE-NCV to find the regions of the brain corresponding to the most discriminative features. The SVM-RFE-NCV and classification algorithms are implemented by using Scikit-learn (Pedregosa et al., 2012).

### Scatter Plot and Correlation Visualization

**Figure 2:**
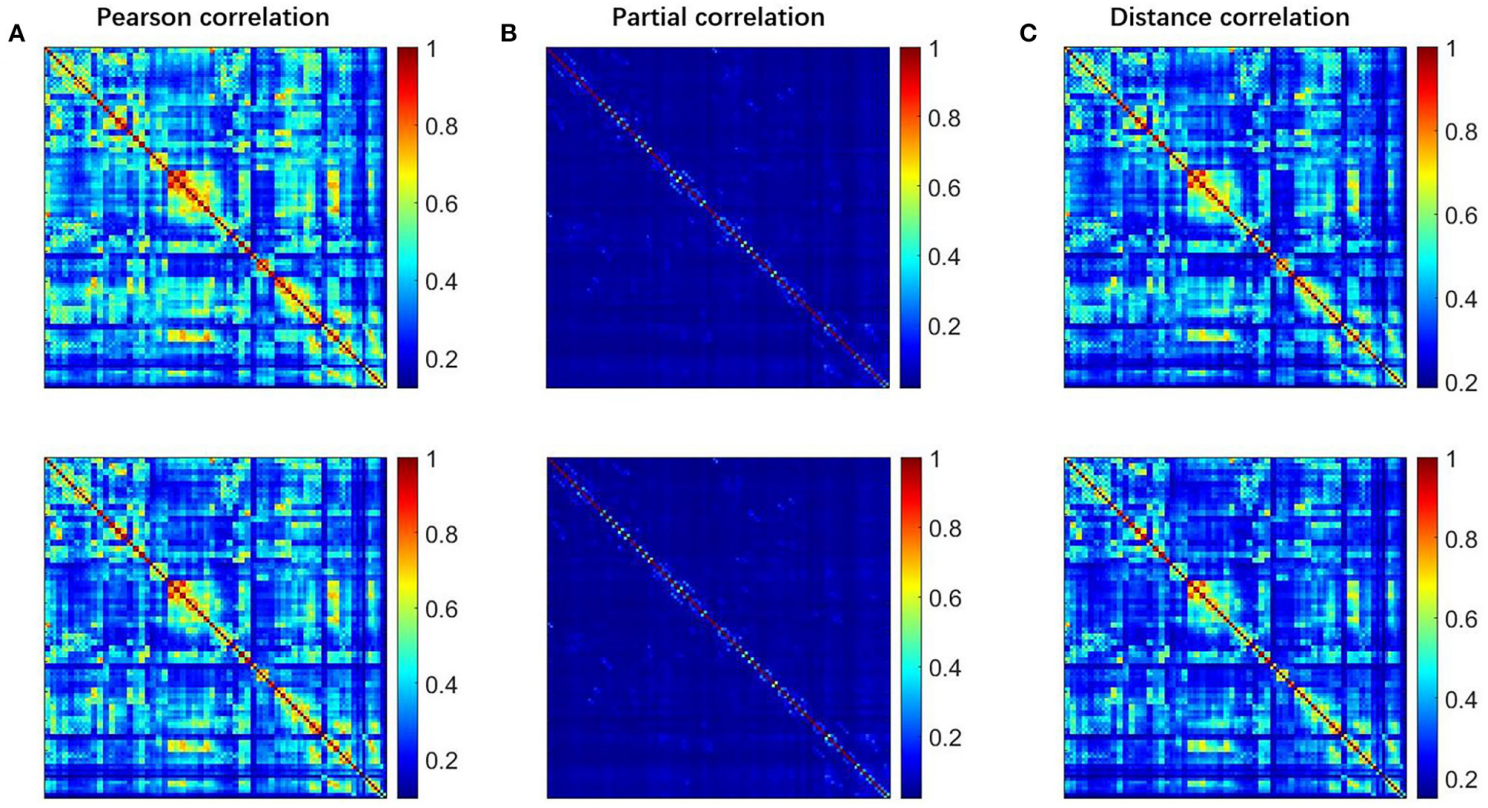
The FC matrices for HCS and the patients with OCD. The first row is for HCS and the second row is for the patients with OCD. **(A)** Pearson correlation, **(B)** partial correlation, and **(C)** distance correlation. FC, functional connectivity; OCD, obsessive-compulsive disorder; HCS, healthy control subject.

**Figure 3:**
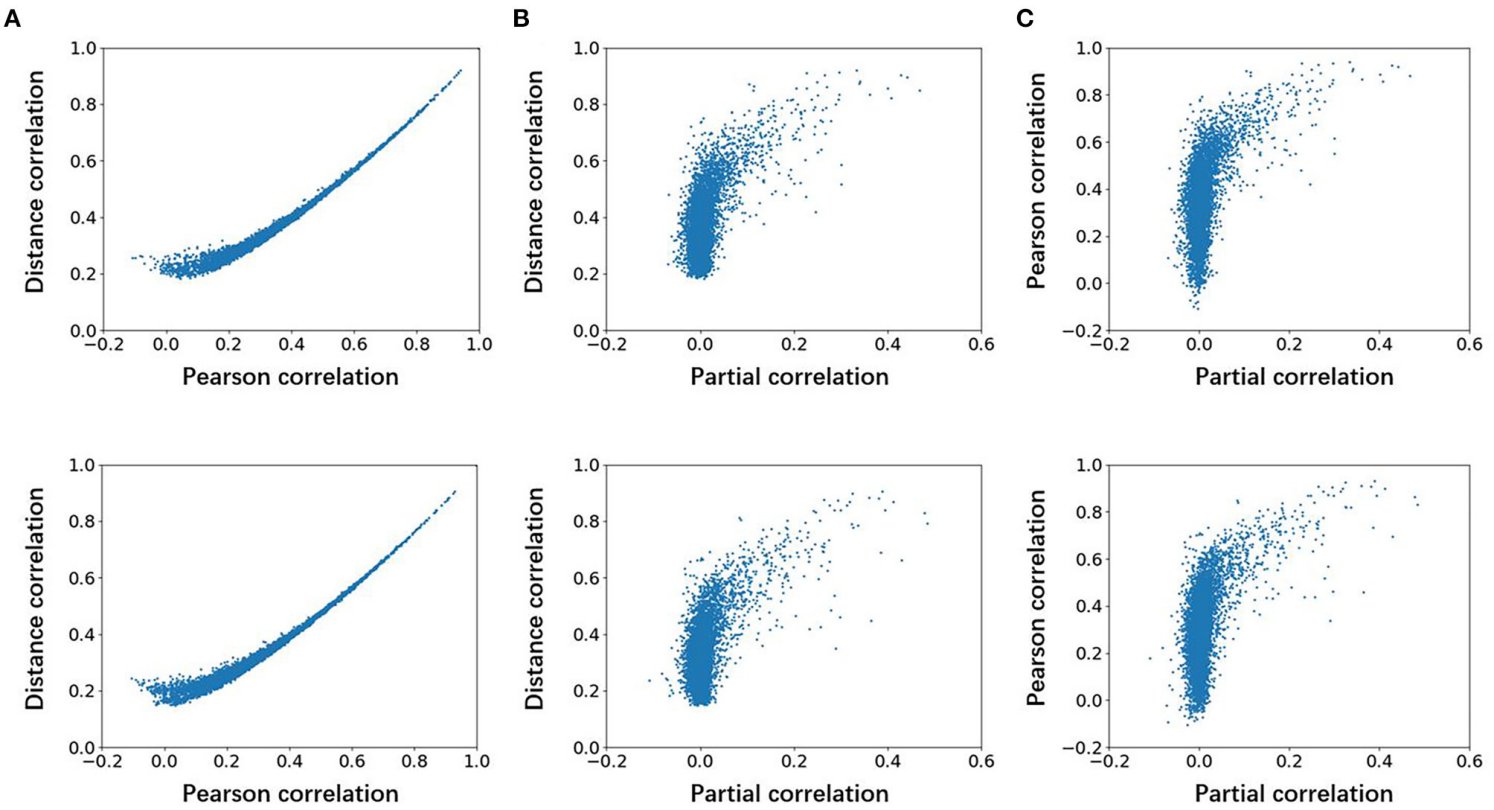
Scatter plots of association among the Pearson, partial, and distance correlations for average functional connectivity across the participants. The first row is for HCS and the second row is for the patients with OCD. **(A)** Distance correlation vs. Pearson correlation, **(B)** distance correlation vs. partial correlation, and **(C)** pearson correlation vs. partial correlation. OCD, obsessive-compulsive disorder; HCS, healthy control subject.

To explore the differences among the Pearson, partial, and distance correlations, we calculated the average functional matrices of the patients and HCS, respectively. Since the distance correlation coefficient ranges from 0 to 1 while the other two range from−1 to 1, we used the unsigned versions of the Pearson and partial correlation coefficients (for example, taking the absolute value of them). The results are shown in Figure 2 and we can see that both the distance and Pearson correlations give similar structures of the functional matrix, while the structure of partial correlation is greatly different from them. To further reflect the similarities and differences among the Pearson, distance and partial correlations, we draw their scatter plots as shown in Figure 3. The values of three coefficients mainly lie in the different intervals: (-0.1, 0.9) for the Pearson correlation coefficients, (-0.1, 0.1) for the partial correlation coefficients, and (0.1, 0.8) for the distance correlation coefficients.

### Choice of FC Method

To find the optimal correlation method, we proceeded in two steps. First, for each correlation method, we used the SVM-RFE-NCV to find the best feature subset that gave the prediction. Second, we compared the performance of each correlation method on the best feature subset.

#### Best Feature Subset

For the SVM-RFE-NCV, the number of optimal features (NOFs) varies with *N*. Table 2 summarizes the changes of accuracy and NOF under the different *N* conditions. For the Pearson correlation, partial correlation, and distance correlation, the performance is the highest when *N* is equal to 5, 8, and 5, respectively. Therefore, the best feature subset of the Pearson correlation and distance correlation was obtained by the SVM-RFE-5CV algorithm. The best feature subset of partial correlation was achieved by the SVM-RFE-8CV algorithm.

**Table 2:** Results of classification by the different number of the features.

**Method**	***N* = 3**	***N* = 4**	***N* = 5**	***N* = 6**	***N* = 7**	***N* = 8**	***N* = 9**	***N* = 10**
**Pearson Correlation**								
Accuracy (%)	85.77	89.68	89.74	88.91	87.31	87.37	85.76	88.89
NOF	108	83	84	233	111	119	101	106
**Partial Correlation**								
Accuracy (%)	80.13	77.82	80.06	79.29	80.71	84.87	81.60	80.83
NOF	896	829	1,489	1,150	971	2,488	1,467	1,531
**Distance Correlation**								
Accuracy (%)	85.06	89.03	93.01	89.87	86.60	88.91	89.80	87.43
NOF	60	91	81	96	107	245	112	412

*NOF, number of features*.

#### Best Correlation Method

**Figure 4:**
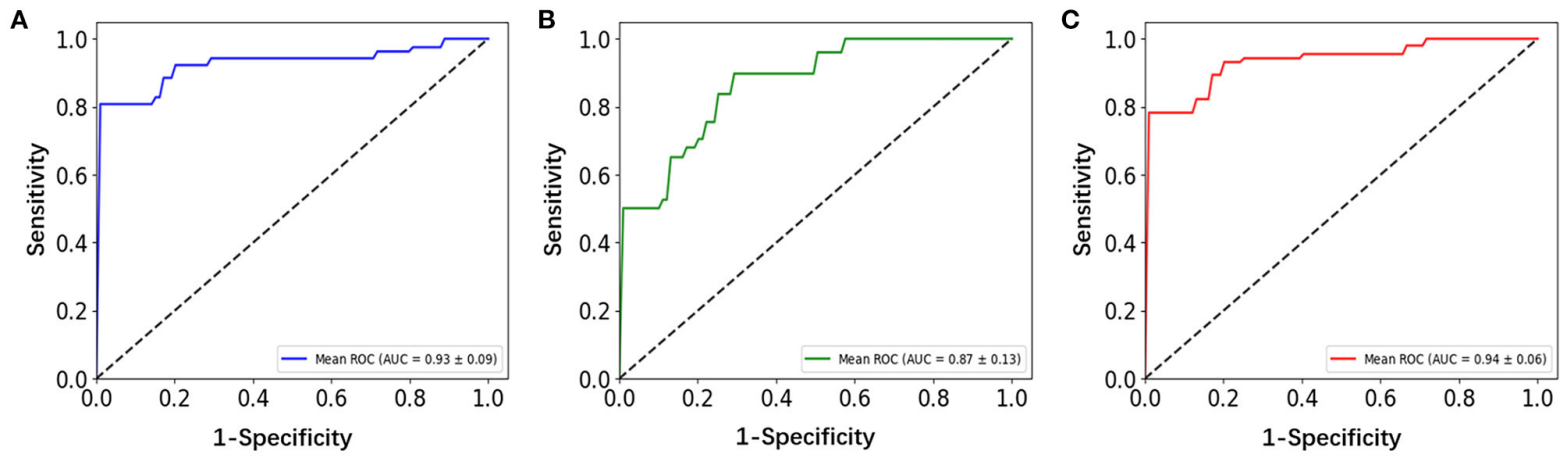
ROC curves assessing Pearson correlation, partial correlation, and distance correlation performance by using SVM. **(A)** Pearson correlation, **(B)** partial correlation, and **(C)** distance correlation. ROC, receiver-operating characteristic; SVM, support vector machine.

Three correlation methods produced a good performance in the classification. Their ROC curves are shown in Figure 4, from which we can see that they exhibit good performance, with AUC values range from 0.87 to 0.94 (*p* < 0.01). The other classification results of the three correlation methods for the patients with OCD and HCS are summarized in Table 3. The distance correlation and Pearson correlation are slightly lower than partial correlation in sensitivity, but distance correlation was the best in accuracy, specificity, and AUC followed by the Pearson correlation and partial correlation. Therefore, in the classification of the OCD and HCS, distance correlation comprehensive performance is the best. Its accuracy, sensitivity, and specificity are 93.01, 89.71, and 95.08% (*p* < 0.01), respectively. The second is Pearson correlation (accuracy = 89.74%, sensitivity = 89.71%, and specificity = 86.62%, *p* < 0.01). For partial correlation, accuracy is 84.87%, sensitivity is 96.21%, and specificity is 75.90% (*p* < 0.01).

**Table 3:** The classification results of the three correlation methods.

**Method**	**Accuracy (%)**	**Sensitivity (%)**	**Specificity (%)**	**AUC**	**NOF**
Pearson correlation	89.74 ± 7.28	89.71 ± 9.22	86.62 ±10.50	0.93 ± 0.09	84 ±46
Partial correlation	84.87 ± 7.09	96.21 ± 5.83	75.90 ± 17.33	0.87 ± 0.13	2488± 1393
Distance correlation	93.01 ± 5.40	89.71 ± 9.22	95.08 ± 7.70	0.94 ± 0.06	81 ± 31

*AUC, area under the receiver-operating characteristic curve; NOF, number of features*.

### Results of Different Classifiers

Through the above analysis, the best discriminative features can be obtained by using the distance correlation and the SVM-RFE-5CV. As stated earlier, the seven classifiers including SVM with linear kernel, MLP, XGBoost, GBDT, GCN, LR-L1, and LR-L2 classifiers were applied to identify these features separately. For SVM, the penalty parameter was set to 1. For LR-L1 and LR-L2, the penalty parameter was set to 0.01. For XGBoost, the learning rate was set to 0.01, the number of gradients boosted trees (n_estimators) to 200, maximum depth of the tree (max_depth) to 5, subsample ratio of the training instance (subsample) to 0.85, the minimum sum of instance weight needed in a child to 2, subsample ratio of the columns when constructing each tree to 0.7, and the other parameters to the default values. For GBDT, the learning rate was set to 0.01, n_estimators to 600, max_depth to 3, subsample ratio to 0.7, the minimum number of samples required to be at a leaf node to 10, the minimum weighted fraction of the total of weights required to be a leaf node to 0.1, and other parameters to the default values. For the GCN and MLP, dropout was set to 0.1, weight decay to 1 × 10^−3^, learning rates to 0.02 and 0.05, number of epochs to 1,000, number of layers to 2, and the numbers of neurons per layer to 128 and 256. The results of classification by 10-CV are given in Table 4. The optimal classification result is achieved *via* SVM for accuracy, sensitivity, specificity, and AUC with values as high as 93.01, 89.71, 95.08%, 0.94 (*p* < 0.01), respectively.

**Table 4:** Results of classification for the data of OCD.

**Classifier**	**Accuracy (%)**	**Sensitivity (%)**	**Specificity (%)**	**AUC**
SVM	93.01 ± 5.40	89.71 ± 9.22	95.08 ± 7.70	0.94 ± 0.06
LR-L1	89.81 ± 6.11	88.46 ± 10.23	91.47 ± 9.25	0.92 ± 0.07
LR-L2	90.58 ± 5.89	89.71 ± 9.22	91.29 ± 7.48	0.94 ± 0.06
GCN	91.41 ± 5.37	89.71 ± 9.22	92.72 ± 7.64	0.95 ± 0.06
MLP	90.64 ± 6.83	89.71 ± 9.22	91.29 ± 7.48	0.94 ± 0.06
XGBoost	85.77 ± 8.85	87.78 ± 11.19	84.84 ± 17.02	0.90 ± 0.12
GBDT	88.97 ± 7.23	86.71 ± 12.72	93.12 ± 9.49	0.94 ± 0.05

*OCD, obsessive-compulsive disorder; SVM, support vector machine; LR-L1, sparse L1 for logistic regression; LR-L2, non-sparse L2 regularization for logistic regression; GCN, graph convolution network; MLP, multilayer perceptron; XGBoost, extreme gradient boosting; GBDT, gradient boosting decision tree; AUC, area under the receiver-operating characteristic curve*.

### Potential Biomarkers From Connectivity Patterns

**Figure 5:**
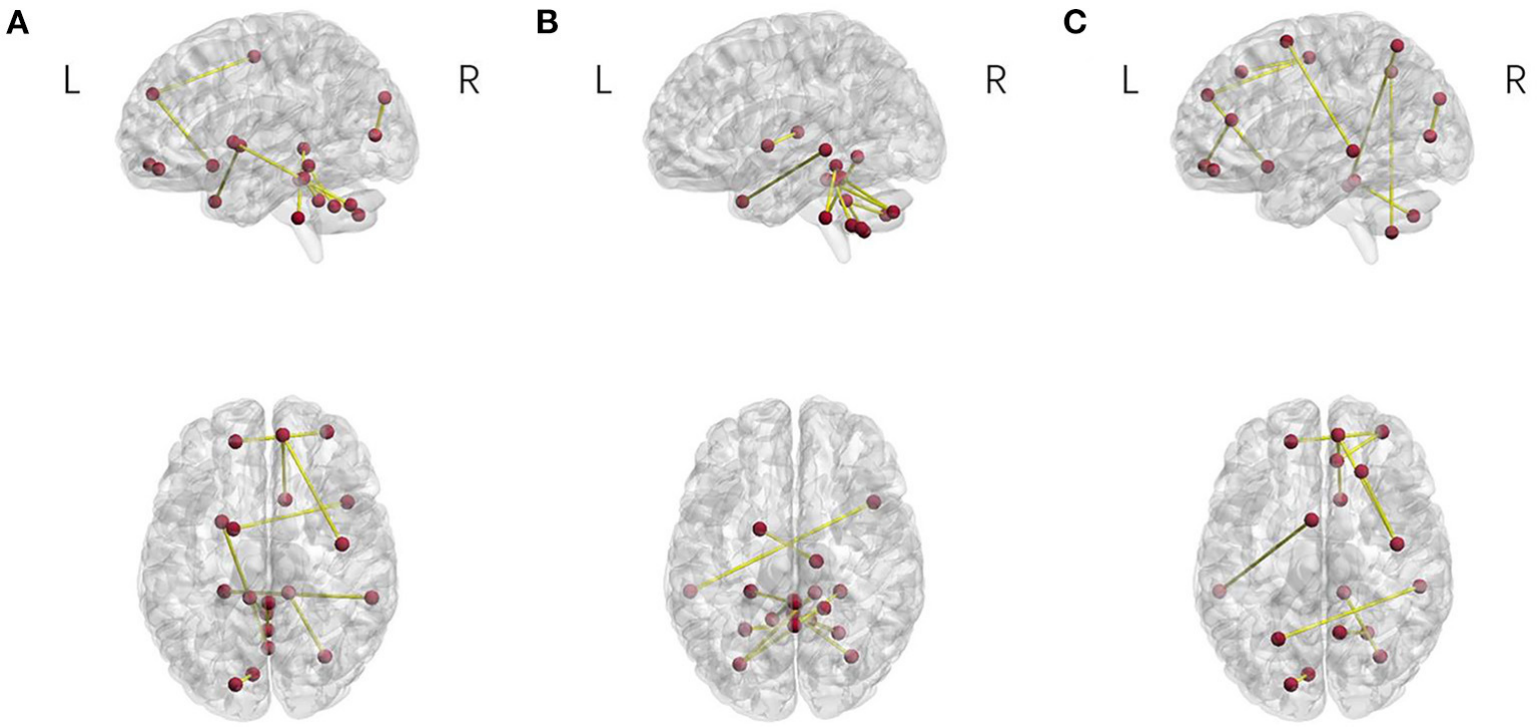
Brain connectivity patterns for the three correlation methods. **(A)** Pearson correlation, **(B)** partial correlation, and **(C)** distance correlation.

To find the regions of the brain that strongly contributed to the discrimination between the patients with OCD and HCS, we selected the top 10 most discriminative features according to the SVM-RFE-NCV method. Specific regions of the brain were then located based on these features. The spatial maps of the regions of the brain (Xia et al., 2013) are shown in Figure 5 and the detailed information is listed in Table 5.

**Table 5:** The most discriminative brain regions.

**Number**	**Pearson correlation**	**Partial correlation**	**Distance correlation**
1	Right precentral gyrus	Left globus pallidus	Right percental gyrus
2	Orbital part of left superior frontal	Right thalamus	Right dorsolateral superior frontal gyrus
3	Orbital part of right middle frontal gyrus	Left middle temporal gyrus	Orbital part of left superior frontal gyrus
4	Right olfactory cortex	Right middle temporal pole	Orbital part of right middle frontal gyrus
5	Medial part of right superior frontal gyrus	Left crus II of cerebellar hemisphere	Left supplementary motor area
6	Left calcarine fissure and surrounding cortex	Right crus II of cerebellar hemisphere	Right olfactory cortex
7	Left superior occipitalgyrus	Right Lobule III of cerebellar hemisphere	Medial part of right superior frontal gyrus
8	Left putamen	Right lobule IV, V of cerebellar hemisphere	Right anterior cingulate and paracingulate gyri
9	Left globus pallidus	Left lobule VIII of cerebellar hemisphere	Left calcarine fissure and surrounding cortex
10	Right middle temporal gyrus	Right lobule VIII of cerebellar hemisphere	Left superior occipitalgyrus
11	Right middle temporal pole	Right lobule VIII of cerebellar hemisphere	Left superior parietal gyrus
12	Right crus II of cerebellar hemisphere	Right lobule IX of cerebellar hemisphere	Right precuneus
13	Left lobule III of cerebellar hemisphere	Left lobule X of cerebellar hemisphere	Right superior temporal pole
14	Right lobule III of cerebellar hemisphere	Right lobule X of cerebellar hemisphere	Right inferior temporal gyrus
15	Left lobule X of cerebellar hemisphere	Lobule I, II of vermis	Left crus II of cerebellar hemisphere
16	Lobule III of vermis	Lobule III of vermis	Left lobule III of cerebellar hemisphere
17	Lobule VIII of vermis	Lobule IV, V of vermis	Right lobule VIII of cerebellar hemisphere
18	Lobule IX of vermis	Lobule X of vermis	
19	Lobule X of vermis		

For Pearson correlation, the most discriminative regions included the right precentral gyrus, orbital part of left superior frontal, orbital part of right middle frontal gyrus, right olfactory cortex, the medial part of right superior frontal gyrus, left calcarine fissure and surrounding cortex, left superior occipital gyrus, left putamen, left globus pallidus, right middle temporal gyrus, right middle temporal pole, right crus II of cerebellar hemisphere, left lobule III of cerebellar hemisphere, right lobule III of cerebellar hemisphere, left lobule X of cerebellar hemisphere, lobule III of the vermis, lobule VIII of the vermis, lobule IX of the vermis, and lobule X of the vermis.

For partial correlation, the most discriminative regions for OCD were composed of the left globus pallidus, right thalamus, left middle temporal gyrus, right middle temporal pole, left crus II of cerebellar hemisphere, right crus II of cerebellar hemisphere, right lobule III of cerebellar hemisphere, right lobule IV of cerebellar hemisphere, right lobule V of cerebellar hemisphere, left lobule VIII of cerebellar hemisphere, right lobule VIII of cerebellar hemisphere, left lobule VIII of cerebellar hemisphere, right lobule IX of cerebellar hemisphere, left lobule X of cerebellar hemisphere, and right lobule X of the cerebellar hemisphere.

For distance correlation, the discriminative regions for OCD primarily consisted of the right percental gyrus, right dorsolateral superior frontal gyrus, orbital part of left superior frontal gyrus, orbital part of right middle frontal gyrus, left SMA, right olfactory cortex, the medial part of right superior frontal gyrus, right anterior cingulate and paracingulate gyri, left calcarine fissure and surrounding cortex, left superior occipital gyrus, left superior parietal gyrus, right precuneus, right superior temporal pole, right inferior temporal gyrus, left crus II of cerebellar hemisphere, left lobule III of cerebellar hemisphere, and right lobule VIII of the cerebellar hemisphere.

## Discussion

The goal of this study is to investigate the potential diagnostic value of the different correlation methods in patients with OCD. We systematically compare the FC matrix-based prediction methods. Our results show that the distance correlation method is optimally followed by the Pearson correlation and partial correlation methods. Besides, a suitable classifier can effectively improve classification performance and it is vital to choose a suitable one. For this reason, we perform many experiments on the multiple classifiers (e.g., LR-L1, SVM). By comparing the different classification results, we found that SVM is the most suitable one in terms of the quantitative results.

We explored the important nodes and connectivity patterns in the network of the brain constructed by the three correlation methods. In these networks, many abnormal areas of the brain and connectivity mentioned in the previous studies about OCD were found including areas in and out of the classical CSTC circuit such as the precentral gyrus and SMA (Ku et al., 2020). These results provide preliminary support for the use of the three correlation methods, especially distance correlation, as promising classification markers for patients with OCD.

Of the three FC methods, distance correlation showed the greatest diagnostic accuracy for discriminating the patients with OCD from HCS. It has been shown that distance correlation directly reflects linear and non-linear correlation in the ROIs. Therefore, the location of the regions of the brain based on distance correlation also showed a considerable research value. For example, the SMA is involved in the planning of the movement. It has been found to involve the compulsion and repetitive behavior of OCD (Gillan et al., 2016). This effect could make the distance correlation method more sensitive to detect dysfunctional neural activity than the other two FC methods. In addition, we can find that the FC matrix based on distance correlation calculation has some intergroup differences. These intergroup differences for the FC matrix may underlie the excellent classification achieved in the current study. Therefore, these ML algorithms were able to identify the patients with OCD and HCS through the FC matrix based on distance correlation calculation. This also provides support for the FC matrix composed of distance correlation calculation as a promising classification marker for OCD.

Pearson correlation was a widely used correlation method, which was generally used to measure the linear relationship between the ROIs. Therefore, its classification performance was lower than distance correlation. In addition, they showed the similarities and differences in the regions of the brain of the Pearson and distance correlation localization. These same regions of the brain had the right precentral gyrus, orbital part of the left superior frontal, and medial part of the right superior frontal gyrus, etc. In this study, they played a critical role in exploring the pathogenesis of OCD. The medial part of the right superior frontal gyrus, corresponding to the left ventral medial prefrontal cortex (vmPFC), has also been found in the disrupted emotion and cognition induced by the symptoms of OCD (Becker et al., 2014; Apergis-Schoute et al., 2017). These different regions of the brain included left putamen, right anterior cingulate, and paracingulate gyri, etc. Among them, the regions of brain (e.g., globus pellidus, putamen.) located by Pearson correlation have great research value (Hibar et al., 2018; Calzà et al., 2019). However, due to the limitation of Pearson correlation measuring linear dependency, some crucial regions of the brain will be ignored. The brain regions located (e.g., anterior cingulate and paracingulate gyri, SMA) by distance correlation can complement and extend it (Ku et al., 2020).

Partial correlation shows good classification performance, although it is lower than the Pearson and distance correlations. It was generally used to exclude the indirect influence of the correlation structure. In other words, it can measure the degree of linear correlation between two regions of the brain without indirect influence from other regions of the brain. Therefore, we can infer that more consideration should be given to the synergies between the multiple regions of the brain in OCD. In addition, we can see that they are mainly distributed in the cerebellum in the regions of the brain defined by partial correlation. In previous studies, the cerebellum also played an important role in the exploration of the pathogenesis of OCD (Zhang et al., 2019). Therefore, we believe that partial correlation has a high potential to explore the influence of the cerebellum on OCD.

In this study, the discriminative regions of the brain are in and out of the CSTC circuit. Previous studies have reported that the orbitofrontal cortex (OFC) play crucial roles in processing reward, negative effect, and, specifically, fear and anxiety in OCD (Kringelbach and Rolls, 2004; Milad and Rauch, 2007). A recent meta-analysis of the voxel-based morphometry (VBM) studies showed decreased gray matter in the bilateral OFCs (de Wit et al., 2014). In fMRI studies, the researchers revealed the white matter abnormalities in OFC (Piras et al., 2013). Furthermore, the anterior cingulate cortex (ACC), putamen, and thalamus have been suggested to play important roles in the previous studies of OCD (Yoo et al., 2007; Zhu et al., 2015; Fan et al., 2017; Hazari et al., 2019). The OCD severity associations have been reported with hypermetabolism in the ACC (Swedo et al., 1989). In this study, consistent with these findings, the OFC, the ACC, putamen, and thalamus displayed a high degree of discriminative ability between the patients with OCD and HCS. These results provide further support for dysfunction in the CSTC circuit in patients with OCD.

In addition, some researchers found that OCD is related to the sensorimotor network (i.e., precentral gyrus/SMA) (Cui et al., 2020). Morein-Zamir et al. (2016) reported that the activation from the regions of the brain within the sensorimotor network in the inhibitory control processes may explain the essence of inhibitory control deficits of OCD. Meanwhile, one recent study indicated that OCD was associated with increased activity in the SMA. With repetitive transcranial magnetic stimulation (rTMS) treatment in SMA, the researcher can observe a reduction in the Y-BOCS score at the 4th week. The reduction in compulsion contributed to the reduction of the global Y-BOCS (Lee et al., 2017). Therefore, these previous results further support this study.

Finally, this study also showed that the cerebellum contributed to distinguish between the patients with OCD and HCS. For example, Miquel et al. (2019) suggested that inhibiting activity in the cerebellar cortex would increase impulsive and compulsive symptomatology. On the other hand, the stimulation of the cerebellar cortex should improve behavioral inhibitory control. Meanwhile, other previous studies reported the existence of disconnectedness in the fronto-striato-limbic community and connectedness between the cerebellar and visual areas in the patients with OCD, which was also related to the clinical symptomatology of OCD (Kashyap et al., 2021).

Despite the encouraging performance achieved, there are still two major limitations in the current research. First, the current study is only evaluated in a small database, which will make the results difficult to generalize. Second, the proposed method uses only single image modality data. Using a variety of modalities can obtain comprehensive features and improve the classification performance of the model. However, the subjects with the multimodal image data in the database are limited. In the future, we will explore the multimodal image data to discriminate the patients with OCD from HCS.

## Conclusion

To conclude, the current experimental results show that it is promising to apply distance correlation for measuring the FC between the ROIs of the brain with contrast to both the traditional Pearson correlation and partial correlation. Besides improving the discrimination performance between the patients with OCD and HCS, the selected biomarkers *via* the SVM-RFE-NCV strategy may provide the potential clinical values for the patients with OCD.

## Data Availability Statement

The raw data supporting the conclusions of this article will be made available by the authors, without undue reservation.

## Ethics Statement

The studies involving human participants were reviewed and approved by the Shenzhen Kangning Hospital. The patients/participants provided their written informed consent to participate in this study.

## Author Contributions

WL, ZP, and CC designed the experiment. CC obtained the funding. LJ and CC collected and preprocessed rs-fMRI data. QL performed the multivariate pattern analysis and completed the first draft of this article. WL, ZP, CC, and QL have finished the final version of this article. All the authors contributed and approved the final version of the article.

## Funding

This study was partly supported by grants from the National Natural Science Foundation of China (61971289 and 31871113), the Shenzhen Fundamental Research Project (JCYJ20170412111316339 and JCYJ20160422113119640), and the Shenzhen-Hong Kong Institute of Brain Science—Shenzhen Fundamental Research Institutions.

## Conflict of Interest

The authors declare that the research was conducted in the absence of any commercial or financial relationships that could be construed as a potential conflict of interest.

## Publisher's Note

All claims expressed in this article are solely those of the authors and do not necessarily represent those of their affiliated organizations, or those of the publisher, the editors and the reviewers. Any product that may be evaluated in this article, or claim that may be made by its manufacturer, is not guaranteed or endorsed by the publisher.
